# MiRNAs regulate oxidative stress related genes via binding to the 3′ UTR and TATA-box regions: a new hypothesis for cataract pathogenesis

**DOI:** 10.1186/s12886-017-0537-9

**Published:** 2017-08-14

**Authors:** Changrui Wu, Zhao Liu, Le Ma, Cheng Pei, Li Qin, Ning Gao, Jun Li, Yue Yin

**Affiliations:** 1 Department of Ophthalmology, the First Affiliated Hospital of Medical School of Xi’an Jiaotong University, Xi’an, Shaanxi Province 710061 China; 2 School of Public Health, Xi’an Jiaotong University Health Science Center, Xi’an, Shaanxi Province 710061 China; 3 Basic Research Center, Affiliated Shaanxi Provincial Tumor Hospital, School of Medicine, Xi’an Jiaotong University, Xi’an, Shaanxi Province 710061 China

**Keywords:** Cataract, Oxidative stress, miRNAs

## Abstract

**Background:**

Age-related cataracts are related to oxidative stress. However, the genome-wide screening of cataract related oxidative stress related genes are not thoroughly investigated. Our study aims to identify cataract regulated miRNA target genes that are related to oxidative stress and to propose a new possible mechanism for cataract formation.

**Methods:**

Microarrays were used to determine the mRNA expression profiles of both transparent and cataractous lenses. The results were analyzed by significance analyses performed by the microarray software, and bioinformatics analysis was further conducted using Molecular Annotation System. The Eukaryotic Promoter Database (EPD) was used to retrieve promoter sequences and identify TATA-box motifs. Online resource miRWalk was exploited to screen for validated miRNAs targeting mRNAs related to oxidative stress. RNAhybrid online tool was applied to predict the binding between significantly regulated miRNAs in cataract lenses and target mRNAs.

**Results:**

Oxidative stress pathway was significantly regulated in cataractous lens samples. Pro-oxidative genes were half up-regulated (11/20), with a small number of genes down-regulated (4/20) and the rest of them with no significant change (5/20). Anti-oxidative genes were partly up-regulated (17/69) and partly down-regulated (17/69). Four down-regulated miRNAs (has-miR-1207-5p, has-miR-124-3p, has-miR-204-3p, has-miR-204-5p) were found to target 3′ UTR of pro-oxidative genes and could also bind to the TATA-box regions of anti-oxidative genes (with the exception of has-miR-204-3p), whilst two up-regulated miRNAs (has-miR-222-3p, has-miR-378a-3p) were found to target 3′ UTR of anti-oxidative genes and could simultaneously bind to the TATA-box regions of pro-oxidative genes.

**Conclusions:**

We propose for the first time a hypothesis that cataract regulated miRNAs could contribute to cataract formation not only by targeting 3′ UTR but also by targeting TATA-box region of oxidative stress related genes. This results in the subsequent elevation of pro-oxidative genes and inhibition of anti-oxidative genes. This miRNA-TATA-box/3′ UTR-gene-regulation network may contribute to cataract pathogenesis.

**Electronic supplementary material:**

The online version of this article (doi:10.1186/s12886-017-0537-9) contains supplementary material, which is available to authorized users.

## Background

Age-related cataract is till now still the dominant cause of blindness worldwide. Although cataract surgery is a satisfying solution for cataract caused vision impairment, this procedure is not easily available in developing countries [1]. There have been studies on the mechanism of age-related cataract aiming to discover new targets for cataract treatment. However, the specific molecular pathway of this disease still merits further investigation. It has been suggested that oxidative stress plays an important role in cataract formation. Lens proteins undergo non-enzymatic, post-translational modification, accumulate fluorescent chromophores and are more susceptible to oxidation along with aging [2], therefore oxidative stress is partially responsible for cataract formation. It has also been reported that oxidation plays a key role in nuclear cataract, but its causal roles in cortical and posterior subcapsular cataracts are substantially less important [3]. Other groups have published gene profiling results from either cataract tissue samples or cell lines [4, 5]. There are also studies on the function of specific genes in cataract formation, particularly oxidative stress related genes, such as *SOD1*, *TXNIP*, etc. [6–8]. Thereupon oxidative stress related gene regulation is worth investigating.

MicroRNAs (miRNAs) are a group of small non-coding RNAs that modulate many pathways related to various diseases. Our previous work has shown that multiple miRNAs are regulated in human cataractous lenses [9]. However, the targets of these miRNAs still warrant further investigation. In this study, we used gene profiling to screen for differentially regulated mRNAs in human cataractous lenses compared with post mortem clear lenses. Then bioinformatics analysis was applied to examine the gene-pathway distribution and the regulation of oxidative stress related genes in cataract lenses. We compared our previously published cataract regulated miRNAs and these differentially regulated mRNAs, screening for miRNA 3′ UTR binding targets that could contribute to cataract pathogenesis. Our previous work indicated miRNAs could bind to the TATA-box region and promote gene transcription as well as 3′ UTR binding-mediated gene silencing [10, 11], so we also screened for TATA-box targets of the miRNAs reported in our previous publication [9]. Our study establishes a miRNA-TATA-box/3′ UTR-gene-regulation network that may contribute to cataract formation.

## Methods

### Human lens epithelium-capsule sample collection

Lens samples were obtained from Department of Ophthalmology, the First Affiliated Hospital of Medical School of Xi’an Jiaotong University. Epithelium-capsule samples were obtained after capsulorrhexis during surgery (15 age-related cataract patients, age ranges from 60 to 68, no other ocular diseases) or obtained from postmortem transparent lenses within 24 h after death (15 donors, age ranges from 58 to 65, no history of ocular diseases). The degrees of lenticular opacification of patients were determined by the Lens Opacities Classification System III (LOCSIII) [12, 13]. The lenticular opacification of postmortem lenses were grade 1 ~ 2 and cataract lenses were grade 4 ~ 6 (Additional file 1: Table S1). There was no significant difference between transparent and cataractous human lens samples respect to the age of donors in two groups (Additional file 1: Table S1, unpaired t test, *p* > 0.05).

### Total RNA preparation

Five samples were randomly pooled into one lens sample to insure the quality and quantity of extracted total RNA. Total RNA of six pooled samples (three cataractous and three transparent samples) was extracted with Trizol reagent (Invitrogen, Shanghai, China) according to the manufacturer’s instructions. The quality of RNA samples was confirmed by measuring OD260/280 ratio using NanoDrop spectrophotometer (Thermo Scientific, Shanghai, China) and the integrity of RNA samples was verified by agarose electrophoresis.

### Microarray analysis

Gene expression profiling was performed for each pooled RNA sample using a Gene Chip-Human Genome Array (HG-U133 Plus 2.0, Affymetrix, Santa Clara, CA) at CapitalBio Corporation (Beijing, China). Gene Chip microarray service of CapitalBio Corporation is certificated by Affymetrix. Microarray processing was performed according to Gene Chip Expression Analysis Technical Manual provided by Affymetrix. In brief, 1 μg of total RNA was used to synthesize double-stranded cDNA. Biotin-tagged complementary RNA (cRNA) was produced using the MessageAmp II aRNA Amplification Kit (Ambion, Austin, TX). The resulting cRNA was fragmented and hybridized to the microarray. After hybridization the GeneChip arrays were washed, stained and then scanned on a GeneChip Scanner 3000 (Affymetrix).

### Data processing and analysis

Microarray raw data were processed at CapitalBio Corporation. Briefly, the hybridization data were analyzed using GeneChip Operating software (GCOS 1.4, Affymetrix) and normalized using a DNA-chip analyzer. Significant Analysis of Microarray software (SAM) was used to identify mRNAs that exhibited significant differences in expression between transparent and cataractous samples (average fold change >2 or <0.5). Results were screened for mRNAs with FDR (false discovery rate)-corrected *p* values < 0.05.

### Bioinformatics analysis

**Fig. 1:**
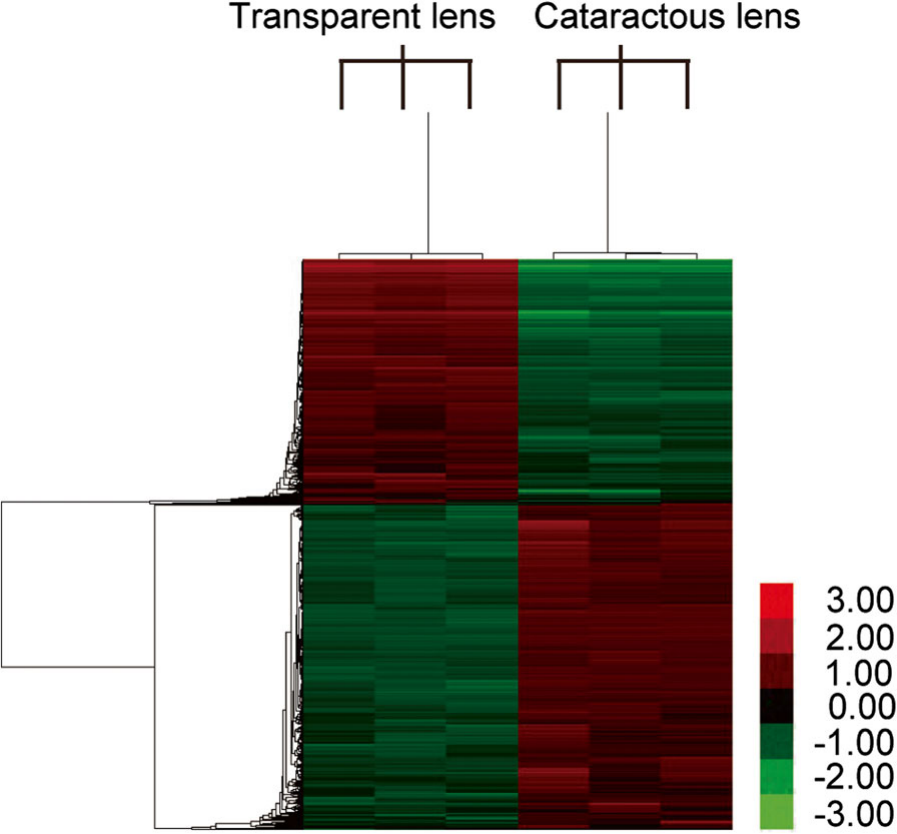
Heatmap shows differentially expressed mRNAs in cataractous lens samples compared with transparent lens samples. Six separate microarray assays were performed to determine the genome-wide mRNA expression in the central epithelium of transparent and cataractous human lenses. Microarray data were processed by CapitalBio Corporation. Heatmap shows differentially expressed mRNAs in cataractous lens samples compared with transparent lens samples. Relative expression value from high to low was shown by gradient of red to green in the heatmap. Colors indicate relative mRNA expression. *Red* and *green* indicate higher or lower expression of mRNAs relative to those in transparent lens samples, respectively. FDR (false discovery rate) adjusted *p*-value <0.05

Bioinformatics analysis was conducted via the online Molecular Annotation System (MAS 3.0) provided by CapitalBio Corporation. Gene symbols of mRNAs with average fold change >2 of <0.5 were uploaded in the MAS 3.0 system for Gene Ontology (GO) and gene-pathway network analysis. Heatmaps were either provided by CapitalBio Corporation (Fig. 1) or generated by using Heatmap illustrator 1.0 according to the user’s manual (Fig. 4) [14]. The Eukaryotic Promoter Database (EPD) [15, 16] was used to retrieve promoter sequences of selected oxidative stress related mRNAs and to identify TATA-box motifs as described in our previous publications [10, 11]. Online resource miRWalk [17, 18] was used to screen for validated miRNAs targeting mRNAs related to oxidative stress. RNAhybrid online tool [19] was applied to predict the binding between significantly regulated miRNAs in cataract lenses in our previous findings [9] and target mRNAs.

### Statistical analysis

Differences between the two groups in microarray data were assessed using FDR (false discovery rate)-corrected *p* values (*p* < 0.05) provided by SAM. Age differences between the two groups were estimated using unpaired t test (*p* < 0.05 was considered statistically significant).

## Results

### Gene profiling in microarrays shows oxidative stress pathway is regulated in cataractous lenses

**Fig. 2:**
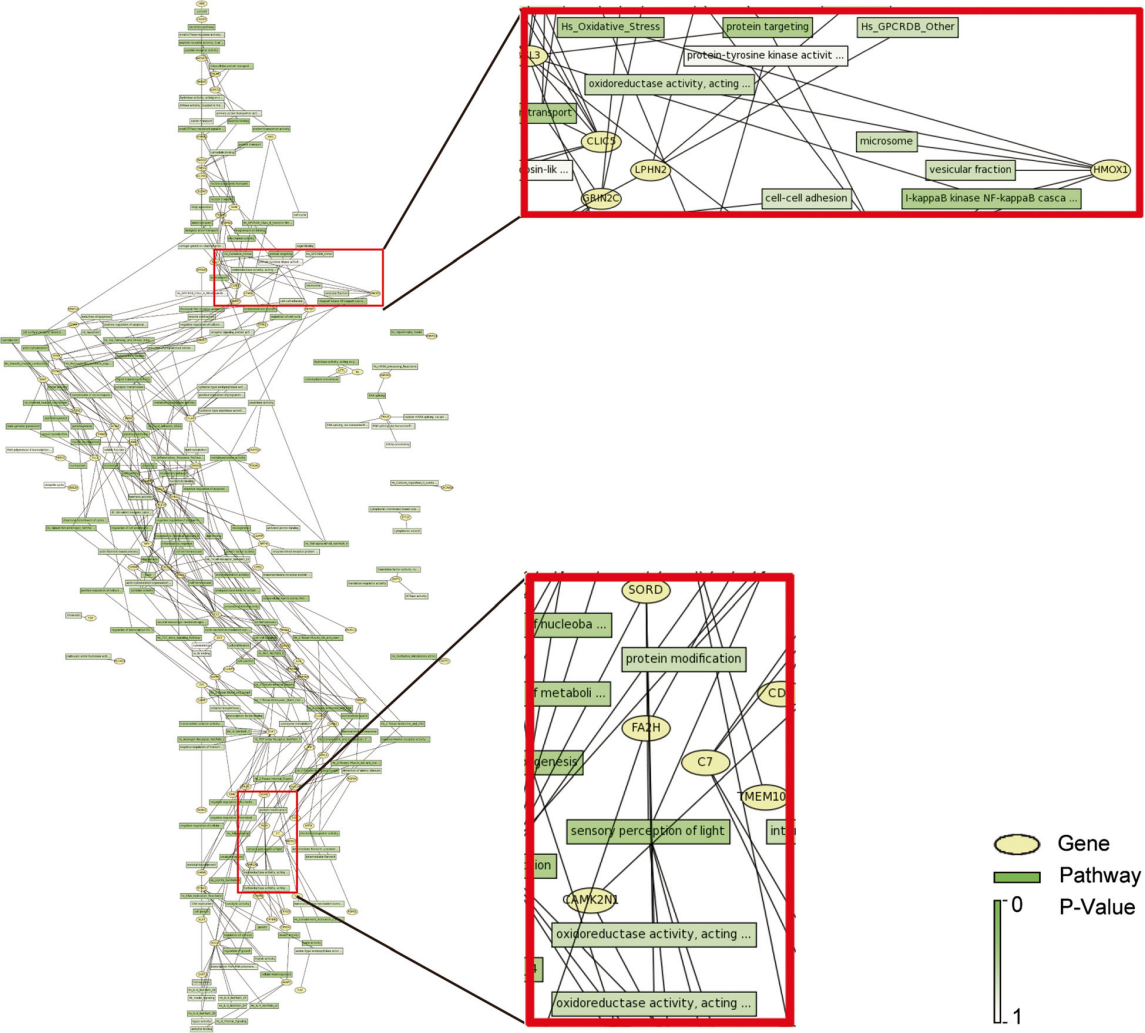
Gene-pathway network graph shows oxidative stress related pathway in the regulated gene-pathway network. Gene symbols of 100 up-regulated genes and 100 down-regulated genes were uploaded to MAS 3.0 system. Then gene-pathway network graph was generated by MAS 3.0 sponsored by CapitalBio Corporation using GenMAPP database. Red-boxed areas show enlarged parts of the gene-pathway network graph related with oxidative stress pathway

**Fig. 3:**
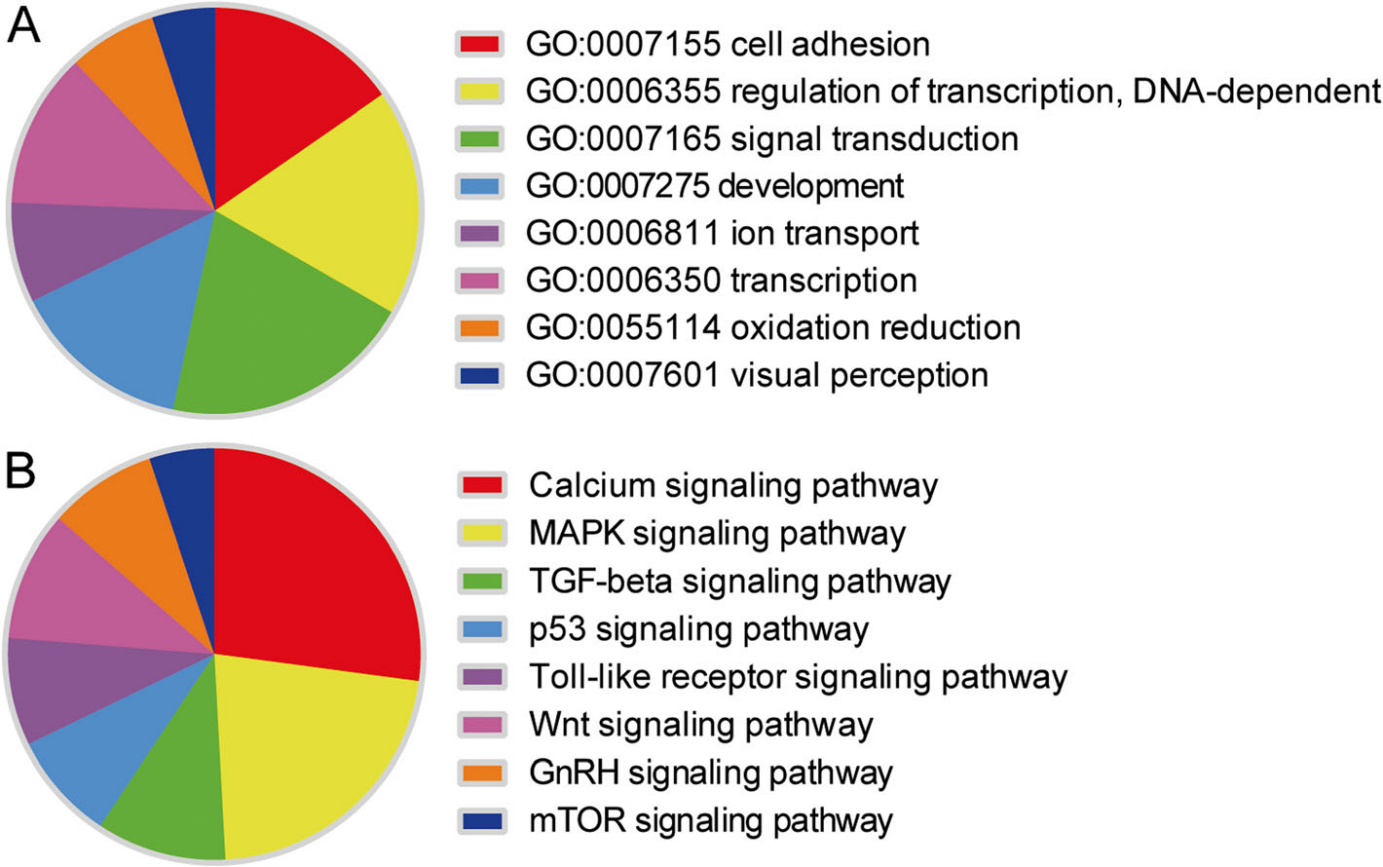
Distribution of cataract-regulated genes in different Gene Ontology (GO) and pathway functional categories. Pie charts show the distribution of cataract-regulated genes in the “biological process” (**a**) and “pathway in Kegg database” (**b**) functional categories of GO and pathway classification. Only the eight most populated classes are shown

Gene profiling was determined by six separate microarray assays, which identified 2223 up-regulated genes and 1829 down-regulated genes in cataractous lenses compared with transparent lenses (average fold change >2 or <0.5) (Fig. 1). Gene symbols of the top 100 up-regulated genes and top 100 down-regulated genes were uploaded to MAS 3.0 system to generate gene-pathway network graph using GenMAPP database, as it was too complex to generate graph by Graphviz if more than 200 gene symbols were included (Fig. 2). It is shown that oxidative stress related pathway was involved in cataract related gene regulation (Fig. 2). The overall distribution of cataract-regulated genes within different categories of the Gene Ontology (GO) classification system (the “biological process” and “pathway in Kegg database” categories) was examined (Fig. 3). Several classes of cataract-regulated genes in the biological process such as signal transduction, transcription, ion transport, etc. were consistent with previous work [4]. Interestingly, oxidation reduction was one of the top eight classes of GO enriched functional categories (Fig. 3a, Additional file 1: Table S2). We also found several pathways that were involved in cataract-mediated gene regulation, such as calcium signaling pathway, MAPK signaling pathway, TGF-beta signaling pathway, etc. (Fig. 3b).

### Expression of oxidative stress pathway related genes in cataractous lenses

**Fig. 4:**
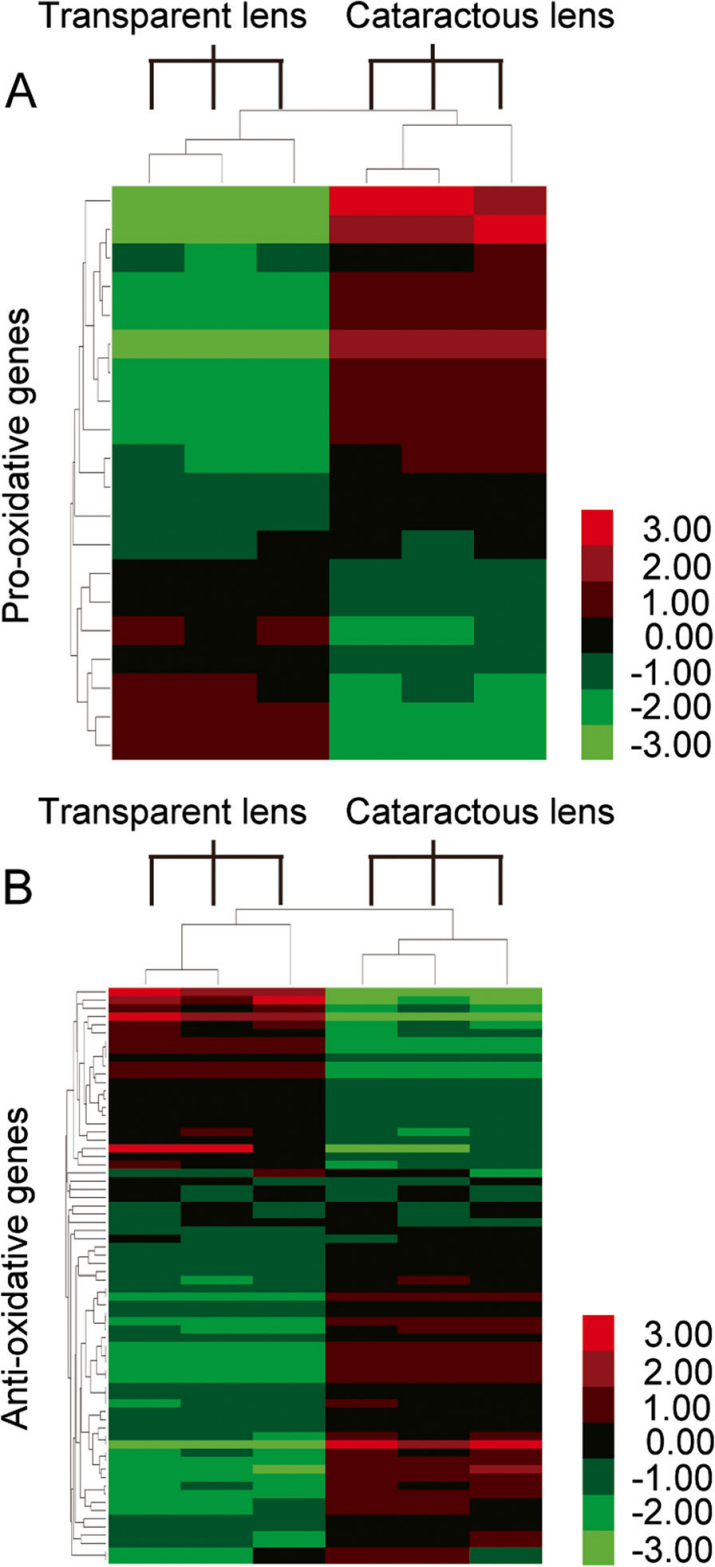
Heatmaps show differentially regulated pro-oxidative or anti-oxidative genes in cataractous lens samples. Heatmaps of selected pro-oxidative genes (**a**) and anti-oxidative genes (**b**) were generated using Heatmap Illustrator 1.0 [14]

To further investigate the regulation of oxidative stress related genes in cataractous lenses, we generated heatmaps consisting only genes related to oxidative stress (Fig. 4). As it is shown there were more significantly up-regulated pro-oxidative genes (11 of 20) than down-regulated genes (4 of 20) in cataractous lenses (Fig. 4a, Additional file 1: Table S3). The rest 5 genes were not significantly regulated. So the oxidative stress related genes were generally up-regulated in cataractous samples. However, there were equal numbers of up-regulated and down-regulated anti-oxidative genes (17 of 69 for both) (Fig. 4b, Additional file 1: Table S3). The up-regulation of anti-oxidative genes was probably due to the response to elevated oxidative stress in cataractous lenses. The down-regulated anti-oxidative genes and up-regulated pro-oxidative genes could lead to the progression of oxidative stress, thus may contribute to cataract formation.

### MiRNAs bind to the TATA-box/3′ UTR of oxidative stress related genes

**Fig. 5:**
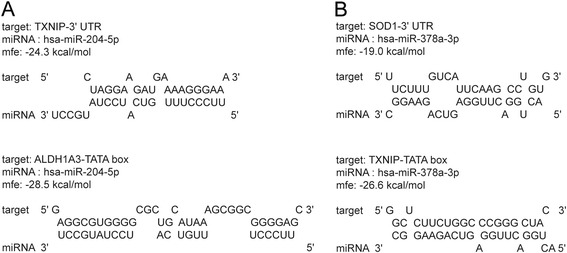
Predicted binding between cataract-regulated miRNAs and the TATA-box region/3′ UTR of oxidative stress related genes. Online resource miRWalk [17, 18] and RNAhybrid [19] was used to predict binding between miR-204-5p/miR-378a-3p and the 3′ UTR of target mRNA *TXNIP/SOD1* (**a** and **b**, *upper part*). The Eukaryotic Promoter Database [15, 16] and RNAhybrid [19] were used to predict binding between miR-204-5p/miR-378a-3p and the TATA-box region of target mRNA *ALDH1A3/TXNIP* promoters (**a** and **b**, *lower part*). mfe: minimum free energy

**Fig. 6:**
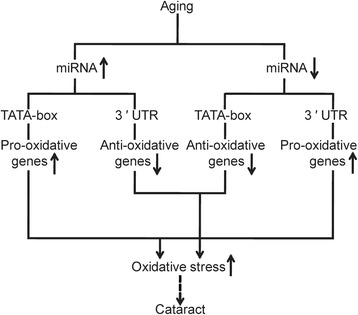
Schematic of hypothesized mechanism of miRNA-regulated oxidative stress related gene expression leading to cataract formation

Since miRNA has been suggested to play a role in cataract formation by our previous work as well as publications of other colleagues [9, 20, 21], we used miRWalk database [17, 18] to screen for miRNA-target oxidative stress related genes. Four down-regulated miRNAs (has-miR-1207-5p, has-miR-124-3p, has-miR-204-3p, has-miR-204-5p) were found to target 3′ UTR of pro-oxidative genes whilst two up-regulated miRNAs (has-miR-222-3p, has-miR-378a-3p) were found to target 3′ UTR of anti-oxidative genes (Tables 1 and 2). To further investigate the part played by these miRNAs, we retrieved the promoter sequences of oxidative stress related genes and predicted the binding between the aforementioned miRNAs and the TATA-box regions of promoters. We found three out of four down-regulated miRNAs could specifically bind to the TATA-box regions of anti-oxidative genes, whilst the two up-regulated miRNAs could target the TATA-box regions of pro-oxidative genes (Table 1). For instance, miR-204-5p could specifically bind to the 3′ UTR of *TXNIP*, which is a regulator of the bioavailability of thioredoxin in the lens, and may promote oxidative stress-induced apoptosis [7]. MiR-204-5p could also bind to the TATA-box region of *ALDH1A3*, which is a member of gene family protecting the eye against cataract formation via detoxification function [8]. Since miR-204-5p was down-regulated in cataractous lenses, it is hypothesized miR-204-5p up-regulates *TXNIP* expression through the reduction of post-transcriptional gene silencing, while down-regulates *ALDH1A3* expression via reduced promoter activation-mediated transcription (Fig. 5a). On the other hand, miR-378a-3p could bind to the 3′ UTR of *SOD1*, a gene preventing hydrogen peroxide -induced oxidative damage to the lens [6]. MiR-378a-3p could also specifically bind to the TATA-box region of *TXNIP*, a pro-oxidative gene [7]. Because miR-378a-3p was up-regulated in cataractous lenses, we propose that miR-378a-3p down-regulates *SOD1* expression via post-transcriptional gene silencing and up-regulates *TXNIP* expression through promoter activation-mediated transcription (Fig. 5b). Our results suggest up-regulated miRNAs down-regulate anti-oxidative genes via 3′ UTR binding, meanwhile up-regulate pro-oxidative genes via TATA-box binding-mediated transcription activation, and it is the opposite for down-regulated miRNAs. This results in the elevation of pro-oxidative genes and inhibition of anti-oxidative genes, which may lead to cataract (Fig. 6).

**Table 1:** Regulated miRNAs in Cataractous Samples and Target mRNA Gene Symbols

miRNA names	regulation	Average fold change^a^	3′ UTR target	TATA-box target
has-miR-1207-5p	down	0.22	CYCS	FTL, MT1E, MT1G, MT1H, MT1M
has-miR-124-3p	down	0.36	CYB5A, CYP1B1	TXN
has-miR-204-3p	down	0.43	CYP1B1	None
has-miR-204-5p	down	0.43	TXNIP	ALDH1A3, TF
has-miR-222-3p	up	2.60	PRDX4, TXN	CYP1A2, CYP1B1
has-miR-378a-3p	up	2.80	SOD1	TXNIP

^a^Average fold change values were means of 3 separate array results (fold change = cataractous lens sample/transparent lens sample)

**Table 2:** Oxidative Stress Related Genes with miRNA Targets in Cataractous Samples

Gene Symbol	Pro-oxidant^(a)^	Anti-oxidant^(a)^	Regulation	Average fold change^(b)^
ALDH1A3	N	Y	down	0.50
FTL	N	Y	down	0.50
MT1E	N	Y	down	0.50
MT1G	N	Y	down	0.32
MT1H	N	Y	down	0.50
MT1M	N	Y	down	0.15
PRDX4	N	Y	down	0.50
SOD1	N	Y	down	0.50
TF	N	Y	down	0.17
TXN	N	Y	down	0.50
CYB5A	Y	N	up	2.00
CYCS	Y	N	up	2.73
CYP1A2	Y	N	up	2.00
CYP1B1	Y	N	up	7.11
TXNIP	Y	N	up	6.36

^(a)^ “Y” = yes; “N” = no

^(b)^ Average fold change values were means of 3 separate array results (fold change = cataractous lens sample/transparent lens sample)

## Discussion

Age-related cataract is believed to be the result of post-translational modification, the accumulation of fluorescent chromophores, increasing susceptibility to oxidation, etc. [2]. Oxidative stress plays an important role in nuclear cataract formation, therefore in this study we exploited genome microarray to determine the genes regulated in cataractous lenses, especially genes related with oxidative stress. Pro-oxidative genes were nearly half up-regulated (11/20), with a small number of genes down-regulated (4/20) and the rest of them with no significant change (5/20). Interestingly, anti-oxidative genes were partly up-regulated (17/69) and partly down-regulated (17/69). The possible explanation of this discrepancy is oxidative stress is not the only cause of nuclear cataract and other mechanism might contribute to this disease as well. Furthermore, there may be a compensation mechanism towards oxidative damage, and the up-regulation of some anti-oxidative genes and the down-regulation of some pro-oxidative genes may act as a response to oxidative stress. Finally, there are limitations in our study such as the limited number of genes selected related to oxidative stress, lack of experimental validations, etc.

MiRNAs have been linked to cataract pathogenesis in studies from us and others as well [9, 20–25]. It would be worth exploring the relationship between these miRNAs and oxidative stress related genes. The classic pathway of miRNA regulated gene expression is via 3′ UTR binding-mediated post-transcription gene silencing. Our previous work indicated miRNAs could also bind to the TATA-box region and act as a gene transcription activator [10, 11]. Therefore, we took advantage of bioinformatics web tools and online resources to screen for targets of cataract regulated miRNAs. In this study, we found cataract regulated miRNAs could contribute to cataract formation not only by targeting 3′ UTR, but also by binding to the TATA-box region of oxidative stress related genes. This resulted in the subsequent elevation of pro-oxidative genes and inhibition of anti-oxidative genes. The elevated level of oxidative stress may lead to cataract formation.

## Conclusions

In conclusion, we propose a hypothesis that this miRNA-TATA-box/3′ UTR-gene-regulation network may contribute to cataract pathogenesis. Our next step would be to validate these aforementioned miRNAs and mRNAs, to certify the target relationship between these miRNAs and their corresponding mRNAs, and to elucidate the specific mechanism of miRNA-TATA-box/3′ UTR-gene-regulation network during cataract formation.

## Additional file

Additional file 1: Table S1. Degrees of Lenticular Opacification Determined by Lens Opacities Classification System III (LOCSIII). **Table S2.** Top 8 Populated Gene Ontology Terms of the Biological Process Category. **Table S3.** Selected Oxidative Stress Related Genes (DOCX 23 kb)

## Abbreviations

cRNA: Biotin-tagged complementary RNA
EPD: The Eukaryotic Promoter Database
FDR: False discovery rate
GO: Gene ontology
LOCSIII: Lens Opacities Classification System III
MAS 3.0: Molecular Annotation System
miRNAs: MicroRNAs
SAM: Significant Analysis of Microarray software
