# Supplementary material for: MiRNAs regulate oxidative stress related genes via binding to the 3′ UTR and TATA-box regions: a new hypothesis for cataract pathogenesis
Source: BMC Ophthalmol. 2017 Aug 14;17:142. doi: 10.1186/s12886-017-0537-9 (PMC5556341; doi:10.1186/s12886-017-0537-9)
Supplement: Additional file 1: Table S1. — Degrees of Lenticular Opacification Determined by Lens Opacities Classification System III (LOCSIII). Table S2. Top 8 Populated Gene Ontology Terms of the Biological Process Category. Table S3. Selected Oxidative Stress Related Genes (DOCX 23 kb) [file 12886_2017_537_MOESM1_ESM.docx]

Table S1. Degrees of Lenticular Opacification Determined by Lens Opacities Classification System Ⅲ (LOCSⅢ)

| **NO. of**  **transparent**  **lenses for microarray** | **Lenticular opacification**  **(Grade 1～2)** | **Age** | **No. of**  **cataractous**  **lenses for microarray** | **Lenticular opacification**  **(Grade 4～6)** | **Age** |
| --- | --- | --- | --- | --- | --- |
| 1 | NO_1_NC_2_C_1_P_1_ | 60 | 1 | NO_4_NC_5_C_2_P_2_ | 63 |
| 2 | NO_1_NC_1_C_1_P_1_ | 60 | 2 | NO_4_NC_5_C_3_P_2_ | 62 |
| 3 | NO_1_NC_2_C_1_P_1_ | 59 | 3 | NO_5_NC_4_C_2_P_2_ | 68 |
| 4 | NO_1_NC_2_C_1_P_1_ | 58 | 4 | NO_5_NC_5_C_2_P_3_ | 60 |
| 5 | NO_1_NC_2_C_1_P_1_ | 65 | 5 | NO_5_NC_6_C_3_P_2_ | 65 |
| 6 | NO_1_NC_2_C_1_P_1_ | 65 | 6 | NO_6_NC_4_C_2_P_3_ | 67 |
| 7 | NO_1_NC_2_C_1_P_1_ | 58 | 7 | NO_6_NC_4_C_2_P_3_ | 61 |
| 8 | NO_1_NC_2_C_1_P_1_ | 59 | 8 | NO_6_NC_4_C_3_P_2_ | 60 |
| 9 | NO_1_NC_2_C_1_P_1_ | 63 | 9 | NO_6_NC_5_C_2_P_2_ | 66 |
| 10 | NO_1_NC_2_C_1_P_1_ | 64 | 10 | NO_6_NC_5_C_2_P_3_ | 64 |
| 11 | NO_1_NC_2_C_1_P_1_ | 65 | 11 | NO_6_NC_5_C_3_P_2_ | 65 |
| 12 | NO_1_NC_2_C_1_P_1_ | 62 | 12 | NO_6_NC_6_C_2_P_2_ | 65 |
| 13 | NO_2_NC_1_C_1_P_1_ | 63 | 13 | NO_6_NC_6_C_3_P_3_ | 66 |
| 14 | NO_2_NC_1_C_1_P_1_ | 65 | 14 | NO_6_NC_6_C_3_P_3_ | 66 |
| 15 | NO_2_NC_2_C_1_P_1_ | 59 | 15 | NO_4_NC_5_C_2_P_2_ | 64 |

Note: There is no significant age difference among cataractous and transparent lens groups (un-paired t test, p>0.05).

Table S2. Top 8 Populated Gene Ontology Terms of the Biological Process Category

| GO Term | Count | p-Value | q-Value | Gene Symbol |
| --- | --- | --- | --- | --- |
| GO:0007155 cell adhesion | 46 | 1.94E-53 | 1.94E-52 | CXCL12;THBS1;CCL2;LOXL2;DST;DST;DST;CLDN1;PDPN;MSLN;PCDH7;COL5A1;CD72;CADM1;TLN2;TNC;BOC;SCARB2;COL4A3;TPBG;DSC1;DSC1;CLCA2;ALCAM;VTN;F5;GPNMB;DCBLD1;SIRPA;MTSS1;SDK1;OPCML;PKP4;AOC3;APBA1;NFASC;FEZ1;MFAP4;EGFL6;NELL1;OMG;SDK2;IGSF11;CDH4;CTNNA2;LSAMP |
| GO:0006355 regulation of transcription, DNA-dependent | 54 | 1.03E-40 | 7.94E-40 | BCL3;ELL3;TGIF1;CEBPD;ELF3;RUNX1;MED31;MNDA;SF1;PRRX1;TMF1;EPAS1;SFPQ;AFF4;TXNIP;JMJD1C;PITX2;TRIM22;FOXP1;ZBTB44;CDCA7;ZMYM2;ZNF207;NR4A2;MLLT3;KLF6;HEY1;TAF5L;LASS6;ETV1;ATF7;TSC22D1;PARP14;EGR1;SALL1;CSDA;GLIS3;ELL2;ING2;ING2;ZNF826;ZNF765;EYA2;GATA3;CAMTA1;VGLL3;MAL;FEZ1;NR2F1;SOHLH2;DACH2;ARNTL2;STAT4;HOPX |
| GO:0007165 signal transduction | 60 | 2.73E-37 | 1.71E-36 | CXCR7;NAMPT;RASSF6;CXCL12;ANKRD1;SFRP1;SF1;S100A11;EPAS1;MAP3K8;IGFBP5;CHRM1;TNFRSF10D;IMPA2;AKAP10;CXCL14;ITPR2;ADRA2A;DAPK1;PPIC;ITPR3;TNC;RPS6KA3;ITPR1;NR4A2;ANGPTL7;DSC1;IGFBP6;GNAL;ALCAM;GPR158;MX1;RPS6KA6;BAIAP2L1;GNG4;RAP2A;PDK3;AKAP9;RTN1;MTSS1;GNAI1;ADRA2C;ING2;WIF1;GNG11;INPP5F;FLJ32810;GPR87;EPS8;MAL;OPRK1;GPR39;MFAP4;NR2F1;VEGFC;ARNTL2;WNT7A;STAT4;SPARCL1;GPR160 |
| GO:0007275 development | 43 | 4.19E-28 | 1.91E-27 | SIK1;TGIF1;RUNX1;SFRP1;PRRX1;EPAS1;PDPN;NTRK2;SEMA4C;PITX2;S100A16;CADM1;FZD8;LIF;HEY1;HMGB3;GPM6B;DIAPH2;SERPINF1;SPRY1;EFNA5;B3GNT5;DONSON;BMP6;EMP1;ST6GAL2;ID2;EYA2;JAG1;WIF1;NDRG4;VEGFC;IGSF10;EGFL6;SOHLH2;ROBO2;FRZB;DACH2;WNT7A;EVI1;LENEP;NGEF;HOPX |
| GO:0006811 ion transport | 24 | 2.06E-22 | 7.64E-22 | SLC34A2;CACNA1A;GRIN2C;SCNN1A;KCNK3;GRIN2A;SLC38A4;SLC5A3;KCNJ4;SLC25A37;KCNE4;CLIC4;SLC39A4;ITPR2;ITPR3;TPCN1;ITPR1;PLLP;CLCA2;GRIA4;RYR3;CLIC5;TRPM8;FXYD6 |
| GO:0006350 transcription | 37 | 2.64E-21 | 8.82E-21 | ELL3;CEBPD;ELF3;MNDA;SF1;SF1;TMF1;SFPQ;AFF4;TXNIP;JMJD1C;FOXP1;TFAP2C;ZBTB44;CDCA7;ZMYM2;NR4A2;MLLT3;KLF6;ETV1;ATF7;TSC22D1;PARP14;SALL1;SEC14L2;CSDA;GLIS3;ELL2;ING2;ZNF826;ZNF765;EYA2;GATA3;CAMTA1;FEZ1;NR2F1;DACH2 |
| GO:0055114 oxidation reduction | 21 | 3.59E-20 | 1.06E-19 | CBR4;LOXL2;CYP1B1;ASPH;JMJD1C;MDH1;NQO1;ALOX5;EGLN3;MAOA;MECR;SQRDL;SRD5A1;DUS2L;DHRS2;BCO2;AOC3;AOC2;SORD;HMOX1;TYRP1 |
| GO:0007601 visual perception | 15 | 2.61E-19 | 7.06E-19 | EPAS1;CYP1B1;LUM;CRYBB3;GJA3;CRYBA1;AOC2;CA4;CRYBA4;CRYGD;CRYBB1;CRYGC;MIP;CRYGA;CRYGB |

Table S3. Selected Oxidative Stress Related Genes

| Pro-oxidative Genes | | Anti-oxidative Genes | |
| --- | --- | --- | --- |
| Gene Symbol | Average Fold Change* | Gene Symbol | Average Fold Change* |
| CYB5A | 2.00 | ALDH16A1 | 1.04 |
| CYCS | 2.73 | ALDH18A1 | 1.17 |
| CYP11A1 | 2.32 | ALDH1A1 | 1.52 |
| CYP1A2 | 2.00 | ALDH1A3 | 0.50 |
| CYP1B1 | 7.11 | ALDH1B1 | 1.61 |
| CYP20A1 | 0.66 | ALDH1L2 | 2.75 |
| CYP26A1 | 2.03 | ALDH2 | 2.04 |
| CYP27A1 | 3.20 | ALDH3A1 | 2.78 |
| CYP27C1 | 4.99 | ALDH3A2 | 1.41 |
| CYP2R1 | 2.18 | ALDH3B1 | 1.46 |
| CYP2U1 | 1.21 | ALDH5A1 | 0.92 |
| CYP39A1 | 0.81 | ALDH6A1 | 0.51 |
| CYP46A1 | 0.40 | ALDH7A1 | 1.64 |
| CYP4V2 | 1.03 | ALDH8A1 | 1.50 |
| CYP51A1 | 0.33 | ALDH9A1 | 2.35 |
| NOX1 | 0.93 | CAT | 1.51 |
| NOX4 | 0.36 | CP | 1.84 |
| POR | 0.39 | FTH1 | 0.99 |
| TXNIP | 6.36 | FTL | 0.50 |
| XDH | 2.71 | GLRX | 2.09 |
|  |  | GLRX2 | 1.87 |
|  |  | GLRX3 | 0.55 |
|  |  | GLRX5 | 1.54 |
|  |  | GPX1 | 3.44 |
|  |  | GPX3 | 2.84 |
|  |  | GPX4 | 1.79 |
|  |  | GPX7 | 1.92 |
|  |  | GPX8 | 3.12 |
|  |  | GSR | 0.50 |
|  |  | GSS | 0.12 |
|  |  | GSTA1 | 1.54 |
|  |  | GSTA4 | 1.07 |
|  |  | GSTK1 | 3.24 |
|  |  | GSTM1 | 1.66 |
|  |  | GSTM2 | 1.64 |
|  |  | GSTM3 | 2.34 |
|  |  | GSTM4 | 2.21 |
|  |  | GSTM5 | 0.86 |
|  |  | GSTO1 | 3.41 |
|  |  | GSTO2 | 1.56 |
|  |  | GSTP1 | 3.59 |
|  |  | GSTT1 | 8.15 |
|  |  | GSTT2 | 2.12 |
|  |  | GSTZ1 | 1.52 |
|  |  | MGST1 | 0.23 |
|  |  | MGST2 | 1.39 |
|  |  | MGST3 | 1.66 |
|  |  | MSRA | 2.14 |
|  |  | MSRB2 | 0.58 |
|  |  | MSRB3 | 0.33 |
|  |  | MT1E | 0.50 |
|  |  | MT1F | 0.41 |
|  |  | MT1G | 0.32 |
|  |  | MT1H | 0.50 |
|  |  | MT1M | 0.15 |
|  |  | MT1X | 0.63 |
|  |  | MT2A | 0.94 |
|  |  | PON2 | 1.22 |
|  |  | PRDX1 | 1.85 |
|  |  | PRDX2 | 1.00 |
|  |  | PRDX3 | 2.05 |
|  |  | PRDX4 | 0.50 |
|  |  | PRDX5 | 1.06 |
|  |  | PRDX6 | 0.40 |
|  |  | SOD1 | 0.50 |
|  |  | SOD2 | 1.71 |
|  |  | SRXN1 | 0.47 |
|  |  | TF | 0.17 |
|  |  | TXN | 0.50 |

*Average fold change values were means of 3 separate array results (fold change=cataractous lens sample signal/transparent lens sample signal).
